# A Comprehensive Review of SSTR-Based Spect and Pet Imaging in Chronic Inflammatory and Immune-Mediated Diseases

**DOI:** 10.3390/jcm14238451

**Published:** 2025-11-28

**Authors:** Shaobo Li, Alex Maes, Tijl Vermassen, Justine Maes, Sylvie Rottey, Christophe Van de Wiele

**Affiliations:** 1 Department of Diagnostic Sciences, Ghent University, 9000 Ghent, Belgium; 2 Department of Fundamental and Applied Medical Sciences, Ghent University, 9000 Ghent, Belgium; 3 Department of Nuclear Medicine, AZ Groeninge, 8500 Kortrijk, Belgium; 4 Department of Medical Imaging and Pathology, KU Leuven, 3000 Leuven, Belgium; 5 Department of Medical Oncology, Ghent University Hospital, 9000 Ghent, Belgium; 6 Cancer Research Institute Ghent, 9000 Ghent, Belgium

**Keywords:** somatostatin receptor, inflammation, vasculitis, sarcoidosis, rheumatoid arthritis, thyroid-associated ophthalmopathy

## Abstract

**Background:** Somatostatin receptors (SSTRs), especially subtype 2 (SSTR2), are increasingly recognized as valuable molecular targets in the imaging of chronic inflammatory and immune-mediated diseases. Their expression on activated immune and stromal cells enables specific, non-invasive detection of inflammatory activity using radio-labeled somatostatin analogs. **Objective:** This review aims to summarize current evidence on SSTR-targeted imaging across a range of chronic inflammatory and immune-mediated diseases, compare its diagnostic value with ^18^F-FDG PET/CT, and discuss biological mechanisms, clinical applications, and remaining challenges. **Methods:** A literature-based narrative review was conducted, integrating preclinical studies, clinical trials, and comparative imaging research involving SSTR PET/SPECT tracers such as ^68^Ga-DOTATATE, ^68^Ga-DOTANOC, ^99^ᵐTc-HYNIC-TOC, and ^111^In-pentetreotide in diseases including vasculitis, sarcoidosis, autoimmune myocarditis, rheumatoid arthritis, and thyroid-associated ophthalmopathy. **Results:** SSTR-targeted imaging has shown promising specificity for inflammatory lesions and provides favorable lesion-to-background contrast, particularly in tissues with high physiological FDG uptake such as the myocardium and brain. In vasculitis and sarcoidosis, SSTR-targeted tracers may complement FDG PET by improving diagnostic confidence and inter-observer consistency in selected small studies. Mechanistically, SSTR2 expression is closely associated with cytokine-driven immune activation, predominantly involving M1 macrophages. However, current evidence remains limited by heterogeneous receptor expression, variable myocardial uptake, and the lack of standardized imaging protocols. **Conclusions:** SSTR-targeted molecular imaging represents a biologically grounded and clinically promising complementary approach for assessing immune-mediated inflammation. Future developments in tracer design, quantitative standardization, and multicenter clinical validation are warranted to establish its role in precision diagnostics.

## 1. Introduction

Chronic inflammatory and immune-mediated diseases are marked by sustained immune activation, progressive tissue destruction, and eventual organ dysfunction [1]. These conditions, including rheumatoid arthritis, sarcoidosis, vasculitis, and myocarditis, often present with systemic and heterogeneous manifestations, contributing to significant morbidity and long-term disability. Despite advances in immunosuppressive and biologic therapies, early and accurate diagnosis remains a major clinical challenge, largely due to non-specific, overlapping symptoms and the fluctuating nature of disease activity. Conventional biomarkers and structural imaging modalities frequently fail to detect subclinical inflammation or reliably predict disease progression, underscoring the urgent need for more sensitive, immune-specific diagnostic approaches [2,3].

Somatostatin receptors (SSTRs) are a family of G-protein-coupled receptors (SSTR1–SSTR5), originally identified in neuroendocrine systems for their regulatory role in hormone secretion and cell proliferation [4]. In the setting of inflammation, accumulating evidence shows that SSTRs, particularly SSTR2 and SSTR5, are significantly upregulated in activated immune and stromal cells, including classically activated (M1) macrophages, CD4^+^ T cells, dendritic cells, synovial fibroblasts, and orbital fibroblasts. This upregulation is driven by pro-inflammatory cytokines such as tumor necrosis factor-α (TNF-α), interferon-γ (IFN-γ), and interleukin-1β (IL-1β), which activate key transcriptional regulators like NF-κB and STAT1, thereby promoting SSTR gene expression [5]. The selective expression of SSTRs in inflamed tissues provides a strong molecular rationale for targeting these receptors in vivo as a surrogate marker of immune activity.

Molecular imaging using radio-labeled somatostatin analogs offers a non-invasive method to visualize and quantify SSTR-expressing immune cells in sites of inflammation. Unlike structural imaging techniques that primarily detect anatomical abnormalities, molecular imaging enables earlier detection of active inflammation, better disease phenotyping, and real-time assessment of treatment response. In contrast to ^18^F-FDG PET, which accumulates in all metabolically active tissues and is prone to high physiological background uptake in organs such as the brain and myocardium, SSTR-targeted imaging may exhibit improved specificity and lesion-to-background contrast, particularly in anatomical regions where FDG uptake can be confounded by physiological activity. Somatostatin receptor-targeted tracers exhibit heterogeneous receptor-binding profiles that may influence their imaging performance across different inflammatory contexts [4]. Among the five receptor subtypes (SSTR1–SSTR5), SSTR2 predominates in activated macrophages and lymphocytes, forming the principal molecular target for most clinical radiotracers. As summarized in Table 1, ^68^Ga-DOTATATE and ^64^Cu-DOTATATE demonstrate high selectivity for SSTR2, favoring their use in diseases with dominant SSTR2 expression such as atherosclerosis and vasculitis. ^68^Ga-DOTANOC binds to SSTR2, SSTR3, and SSTR5, potentially enhancing detection in disorders with heterogeneous immune cell composition like sarcoidosis or rheumatoid arthritis. ^68^Ga-DOTATOC exhibits predominant affinity for SSTR2 with minor binding to SSTR5, whereas SPECT tracers such as ^99m^Tc-HYNIC-TOC and ^111^In-DOTA-JR11 maintain dual or subtype-selective binding profiles. Understanding these receptor-binding differences is essential for selecting appropriate tracers and interpreting imaging results across diverse inflammatory diseases.

In recent years, a growing number of studies have investigated the use of SSTR-targeted imaging in a variety of chronic inflammatory conditions. These techniques have demonstrated potential for detecting subclinical inflammation, distinguishing active from inactive disease, and guiding immunosuppressive treatment decisions. However, despite these advances, no comprehensive review has yet synthesized the biological rationale, tracer mechanisms, and disease-specific performance of SSTR imaging across inflammatory contexts. In addition, direct comparisons between SSTR-targeted tracers and ^18^F-FDG remain scattered and underexplored.

This review aims to fill this gap by providing a narrative overview of SSTR-based molecular imaging in chronic inflammatory diseases, ranging from receptor biology to tracer characteristics, imaging performance in specific diseases, and head-to-head comparisons with FDG. By consolidating current evidence, we hope to clarify the clinical potential of SSTR imaging and identify key directions for future research and clinical translation. This narrative review focuses on chronic inflammatory disorders rather than acute inflammation or infection. To identify relevant literature, a structured search was conducted in the PubMed, Scopus, and Embase databases using combinations of the terms “somatostatin receptor”, “SSTR”, “single-photon emission computed tomography (SPECT)”, “positron emission tomography (PET)”, and “inflammation”. Only original human studies and review articles published in English were considered. Conference abstracts, editorials, and case reports with fewer than five patients were excluded.

### 1.1. Atherosclerosis

Atherosclerosis is a chronic, systemic disease characterized by the accumulation of fatty and fibrous material in the intimal layer of arteries. It affects multiple vascular territories including coronary, cerebral, large, and peripheral arteries, and remains a leading cause of morbidity and mortality worldwide [6]. Despite advances in preventive strategies and therapeutic interventions, many patients still experience acute events such as myocardial infarction or stroke. Current diagnostic approaches including blood biomarkers, vascular ultrasound, angiography, and intravascular imaging are limited in detecting early-stage or subclinical inflammation. Therefore, there is an urgent clinical need for noninvasive, sensitive imaging techniques to assess disease activity and plaque vulnerability.

Chronic inflammation is a hallmark of progressive atherosclerosis. Macrophages play a central role by infiltrating the intima, internalizing lipids, and producing proinflammatory cytokines, thus promoting plaque formation and instability. Among these immune cells, proinflammatory M1 macrophages are of particular important as they express high levels of somatostatin receptor subtype 2 (SSTR2) [7]. This expression pattern provides a biological rationale for using SSTR-targeted molecular imaging to assess active vascular inflammation.

Rinne et al. performed ^68^Ga-DOTATATE autoradiography, immunohistochemistry and flow cytometry in apolipoprotein E-deficient mice and identified a strong correlation between radioligand-uptake and the degree of inflammatory cell infiltration [8]. Apolipoprotein E plays a key role in lipid metabolism and transportation and its deficiency results in hyperlipidemia and atherosclerosis Similar results were obtained by Meester et al. using the SSTR2 antagonist ^111^In-DOTA-JR11 and single photon emission tomography, demonstrating high-lesion to background ratios in murine plaques [9].

Mojtahedi et al. applied ^68^Ga-DOTATATE PET/CT to assess vulnerable or fibrotic plaques in the coronary arteries of 44 patients with neuroendocrine tumors [10]. Seven coronary segments per patient were evaluated, and tracer uptake showed a significant correlation with plaque progression based on Hounsfield unit analysis (*p* = 0.0026), supporting its potential in identifying subclinical disease. Similarly, Rominger et al. previously investigated ^68^Ga-DOTATATE uptake in the left anterior descending artery (LAD) among 70 oncologic patients undergoing PET/CT [11]. Tracer uptake was detectable in all patients and significantly correlated with the presence of calcified plaques (r = 0.34, *p* < 0.01), prior vascular events (r = 0.26, *p* < 0.05), and male sex (r = 0.29, *p* < 0.05). These results reinforce the association between ^68^Ga-DOTATATE signal intensity and established cardiovascular risk factors, highlighting its potential role in coronary risk stratification.

In a study by LI et al. involving 16 consecutive patients, ^68^Ga-DOTATATE uptake in large vessels was compared with ^18^F-FDG PET, calcified plaques (CPs), and cardiovascular risk factors [12]. ^68^Ga-DOTATATE uptake was detectable in the left anterior descending artery (LAD) in all patients. The target-to-background ratio (TBR) significantly correlated with the presence of CPs (r = 0.34, *p* < 0.01), prior vascular events (r = 0.26, *p* < 0.05), and male sex (r = 0.29, *p* < 0.05). Notably, these correlations mirrored those of CPs, which were also linked to age and hypertension, further supporting the potential role of SSTR imaging in vascular risk stratification.

A foundational study by Tarkin et al. further confirmed the value of ^68^Ga-DOTATATE PET [13]. In vitro, SSTR2 gene expression was shown to be restricted to proinflammatory M1 macrophages, with radioligand binding localized to CD68-positive macrophage-rich regions of carotid plaques. In vivo, SSTR2 mRNA levels in carotid tissue correlated strongly with PET signal intensity (r = 0.89; *p* = 0.02). Clinically, among 42 patients with atherosclerosis, ^68^Ga-DOTATATE PET was able to differentiate culprit from non-culprit arteries in both acute coronary syndrome and transient ischemic attack/stroke cohorts. Compared to ^18^F-FDG, which suffered from myocardial spillover in cardiac imaging, ^68^Ga-DOTATATE may demonstrate higher specificity for vascular inflammation and clearer lesion delineation.

Further advances have been made using PET/MRI in combination with alternative radiotracers. Pedersen et al. performed ^64^Cu-DOTATATE PET/MRI in 10 patients with carotid atherosclerosis and evaluated 62 plaque segments [14]. Tracer uptake was significantly higher in symptomatic plaques and showed strong correlation with CD163 expression, a marker of alternatively activated macrophages (M2-like), suggesting this modality can capture both pro- and anti-inflammatory activity within lesions. Finally, in a study by Malmberg et al., ^64^Cu-DOTATATE demonstrated significantly higher uptake in arterial plaques than ^68^Ga-DOTATOC in a cohort of 60 patients [15]. Moreover, ^64^Cu-DOTATATE uptake was strongly associated with cardiovascular risk scores (Framingham), both in SUVmax (r = 0.4; *p* = 0.004) and TBR (r = 0.3; *p* = 0.04), whereas ^68^Ga-DOTATOC did not show such correlations. These results indicate that ^64^Cu-DOTATATE may provide improved sensitivity and a more accurate representation of atherosclerotic disease burden.

### 1.2. Sarcoidosis

Sarcoidosis is a chronic, multisystem granulomatous disease of unknown etiology that can affect virtually any organ system. Over 90% of patients present with intrathoracic involvement, particularly in the lungs and mediastinal lymph nodes, although the skin, eyes, and other organs may also be affected [16]. Among these manifestations, cardiac sarcoidosis (CS) is one of the most severe complications and may result in life-threatening arrhythmias or progressive heart failure [17]. Timely and accurate diagnosis remains challenging due to disease’s heterogeneous presentation.

At the cellular level, sarcoid granulomas are composed of macrophages, epithelioid cells, and multinucleated giant cells—all of which have been shown to express SSTR2 [18]. This molecular profile enables the use of SSTR-targeted imaging agents, such as ^99^ᵐTc-HYNIC-TOC, ^111^In-pentetreotide and ^68^Ga-DOTANOC, to visualize active inflammation. Compared with conventional ^67^Ga-scintigraphy, SSTR-based imaging offers faster acquisition times, improved spatial resolution, and higher lesion-to-background contrast, making it a promising approach for evaluating disease extent and therapeutic response.

In a study by Piotrowski et al., ^99m^Tc-HYNIC-TOC scintigraphy was performed in 32 patients with sarcoidosis and combined with conventional and novel biomarkers [19]. Notably, patients with positive tracer uptake had significantly elevated levels of 8-isoprostane (8-IP) in exhaled breath condensate (19.1 ± 19.8 vs. 5.4 ± 3.5 pg/mL, *p* = 0.02). Although other markers such as serum ACE and BALF lymphocyte percentages trended higher in the positive group, statistical significance was not reached. Nonetheless, a moderate correlation was observed between uptake ratio and ACE levels (r = 0.44, *p* = 0.041), supporting the potential clinical utility of combining molecular imaging with non-invasive biomarkers for activity monitoring.

Further comparative evidence comes from a prospective study by Kwekkeboom et al., in which ^111^In-pentetreotide SRS was evaluated against ^67^Ga-scintigraphy in 18 patients with biopsy-proven sarcoidosis [20]. SRS detected abnormalities in all patients and identified a greater proportion of clinically involved sites (83%) compared to gallium imaging (65%). Importantly, SRS maintained higher sensitivity even in patients receiving corticosteroid therapy (82% vs. 59%), highlighting its reliability in immunosuppressed populations.

Several clinical studies have demonstrated the potential utility of SSTR imaging in systemic sarcoidosis. Nobashi et al. compared ^68^Ga-DOTATOC PET/CT with conventional ^67^Ga-scintigraphy in a cohort of 20 patients with histologically or clinically confirmed sarcoidosis [21]. DOTATOC-PET/CT exhibited a higher detection rate (95% vs. 85%), identified more lesions—especially in lymph nodes, ocular tissues, and skeletal muscles—and showed significantly greater involvement of mediastinal nodal regions (*p* < 0.0001). Whole-body active lesion volume derived from PET was also moderately correlated with serum ACE levels (*p* = 0.64, *p* = 0.0044), suggesting potential for disease activity assessment.

Cardiac sarcoidosis presents additional imaging challenges, particularly in differentiating myocardial from extracardiac involvement. In a prospective study involving 13 patients with suspected CS who had prior positive ^18^F-FDG PET scans, ^68^Ga-DOTATATE PET was performed approximately 37 days later [22]. Abnormal myocardial uptake was noted in seven patients (four definite, three probable), while six cases were negative. In contrast, ^18^F-FDG showed cardiac uptake in all patients, albeit with variable patterns. Importantly, mediastinal or hilar nodal uptake was observed in 46% of cases and showed 100% concordance between DOTATATE and FDG, whereas myocardial uptake concordance was only 54%. Ex vivo histology confirmed weak SSTR2 expression within myocardial granulomas but not in surrounding tissue, possibly explaining the limited myocardial sensitivity.

In a comparative pilot study of 19 patients with suspected CS, ^18^F-FDG PET/CT produced inconclusive results in 58% of cases, while ^68^Ga-DOTANOC PET/CT yielded definitive findings in all patients [23]. Diagnostic accuracy was notably higher with DOTANOC (100% vs. 79%), and interobserver agreement was better (κ = 0.46 vs. 0.27), underscoring the value of SSTR-based imaging in reducing physiological background noise and improving diagnostic clarity.

Lapa et al. further explored the feasibility of ^68^Ga-DOTATATE PET/CT in 15 patients with systemic sarcoidosis and suspected cardiac involvement by comparing it to cardiac magnetic resonance imaging (CMR) [24]. Using the AHA 17-segment model, 27 myocardial segments were identified as positive by PET versus 29 by CMR, yielding an overall concordance rate of 96.1%. SSTR-PET was positive in 7 patients, and in most PET-negative but CMR-positive cases only minor involvement or no disease progression was observed during follow-up. These findings suggest that SSTR imaging may complement CMR in assessing cardiac sarcoidosis and could help identify clinically significant inflammation.

### 1.3. Myocarditis

Myocarditis encompasses a group of inflammatory cardiomyopathies characterized by immune-mediated injury to myocardial tissue. This includes entities such as cardiac sarcoidosis and post-transplant myocarditis, both of which, although relatively rare, are associated with significant morbidity and mortality. Although relatively rare, these conditions are associated with high morbidity and mortality. The early stages of myocarditis may present with subtle or non-specific symptoms, making timely diagnosis particularly challenging. Traditional diagnostic modalities such as cardiac magnetic resonance imaging (CMR) and endomyocardial biopsy (EMB) are helpful but limited by sensitivity, invasiveness, and inter-observer variability [25]. As such, there is a growing interest in non-invasive molecular imaging tools, including SSTR to detect early inflammatory activity and guide clinical management.

The biological rationale for SSTR-based imaging in myocarditis stems from the expression of somatostatin receptors, particularly SSTR2, on activated lymphocytes and macrophages—key effector cells in immune-mediated cardiac inflammation [26]. Post-transplant rejection involves infiltration of activated immune cells into myocardial tissue. These immune cell populations express SSTRs, providing a molecular target for radio-labeled somatostatin analogues. In fact, SSTRR expression on CD68-positive macrophages and multinucleated cells in myocarditis was recently proven in a series of explant-proven myocarditis (n = 5) and giant-cell myocarditis (n = 11) by Polte et al. [27]. Inversely, in eight multi-organ donors without signs of myocardial inflammation or scarring, no SSTR2 expressing cells were present. By visualizing areas of receptor overexpression, SSTR imaging offers a promising, non-invasive approach to detect early inflammation and monitor disease progression.

Although acute myocarditis is not a chronic inflammatory disorder per se, it shares key immune-mediated mechanisms—particularly macrophage- and T-cell-driven inflammation—and thus provides important insights into the role of SSTR imaging in cardiac immune inflammation. Boursier et al. performed ^68^Ga-DOTATOC myocardial gated PET in a series of 14 patients suffering from acute myocarditis as proven by magnetic resonance imaging and observed an increase myocardial tracer uptake in all patients with an SUVmax ratio of myocardial uptake/blood pool activity exceeding 2.18 in all patients [28]. Larive et al. analyzed factors associated with ^68^Ga-DOTATOC uptake on scans derived from 178 oncological patients, 31 of which were known to suffer from acute myocarditis [29]. Myocardial tracer uptake was apparent in all 31 patients. In the context of transplant-related myocarditis, ^111^In-pentetreotide somatostatin receptor scintigraphy has shown promise as an early marker of cardiac allograft rejection. In a feasibility study by Aparici et al. involving 10 heart transplant recipients who underwent 13 imaging sessions alongside EMB, tracer uptake was visually assessed and quantified using the heart-to-lung ratio (HLR) [30]. Intense or moderate uptake (HLR > 1.6) was noted in 8 scans, three of which corresponded to acute rejection confirmed by biopsy (ISHLT grade 3A/4). Notably, five other scans with elevated uptake showed either mild or no rejection on initial biopsy but progressed to clinically significant rejection within one week. In contrast, scans with low uptake (HLR < 1.6) were associated with absence of rejection on both initial and follow-up histology. These findings suggest that SSTR scintigraphy may help detect early immune activation prior to overt myocyte injury and histological changes, offering a valuable diagnostic window ahead of EMB results. Moreover, its relatively short imaging time (4 h) enhances practicality in clinical settings.

### 1.4. Rheumatoid Arthritis

Rheumatoid arthritis (RA) is a chronic systemic autoimmune disorder primarily affecting synovial joints [31]. The disease is characterized by persistent synovial inflammation, leading to progressive cartilage and bone destruction, functional impairment, and reduced quality of life [32]. Accurate assessment of inflammatory activity is essential for guiding therapeutic decisions and evaluating treatment efficacy. While conventional imaging modalities such as ultrasound and magnetic resonance imaging (MRI) are commonly used, their utility may be limited in the assessment of systemic disease burden and in longitudinal monitoring. Molecular imaging techniques targeting inflammatory markers, such as SSTRs, have therefore attracted interest as complementary tools for evaluating disease activity in RA.

SSTRs are expressed on several cell types involved in RA pathogenesis. Activated endothelial cells and infiltrating lymphocytes within the inflamed synovium have been shown to express high levels of SSTRs, particularly SSTR2 [33]. Additionally, synovial fibroblasts, which contribute to joint destruction through secretion of proteolytic enzymes and proinflammatory cytokines, also express SSTR1 and SSTR2. Furthermore, Reubi et al. found a high expression of SSTR on the veins in the synovium of patients suffering from rheumathoid arthritis suggesting that somatostatin may also act through these venous receptors to modulate the inflammatory process [34]. These patterns of expression provide the biological foundation for the application of SSTR-targeted imaging in RA, potentially enabling the visualization of active inflammation at the molecular level.

In a pilot study conducted by Anzola et al., the radio-labeled somatostatin analogue ^99m^Tc-EDDA/tricine-HYNIC-tyr(3)-octreotide (^99m^Tc-EDDA/HYNIC-TOC) was used to assess inflammatory activity in patients with RA and Sjögren’s syndrome (SS) who were refractory to conventional treatment [35]. The results showed increased radiotracer uptake in all affected joints, as well as in 12 of 18 salivary glands, indicating active immune-mediated inflammation. Following treatment with infliximab, a significant reduction in joint uptake was observed, while uptake in the salivary glands remained largely unchanged. These findings suggest that SSTR-based scintigraphy may be useful for monitoring therapeutic response in joint inflammation, although its sensitivity for glandular involvement appears limited.

In another study by Vanhagen et al., the in vivo and in vitro expression of SSTRs in RA was evaluated [36]. Fourteen patients with RA underwent scintigraphic imaging using ^111^In-DTPA-D-Phe1-octreotide. A total of 274 clinically swollen joints were assessed, among which 207 joints (76 percent) showed focal tracer uptake, consistent with active inflammation. Additionally, autoradiographic analysis of synovial membrane samples using ^125^I-Tyr3-octreotide demonstrated specific SSTR binding, further supporting the in vivo imaging findings. This study provided evidence of both functional imaging detection and histological validation of SSTR expression in inflamed synovial tissue.

### 1.5. ANCA

Anti-neutrophil cytoplasmic antibody (ANCA)–associated vasculitis (AAV) comprises a group of rare but serious systemic small-vessel vasculitis, including granulomatosis with polyangiitis (GPA), microscopic polyangiitis (MPA), and eosinophilic granulomatosis with polyangiitis (EGPA) [37]. Recent epidemiological studies have reported a prevalence of approximately 300 to 421 cases per million population, representing a notable increase likely attributable to improved survival, enhanced disease recognition, and refined classification criteria [38]. The key pathological features of AAV include inflammation of small vessel walls, endothelial injury, and secondary tissue damage, most commonly involving the respiratory tract and kidneys. Despite the widespread use of glucocorticoids and immunosuppressive agents, disease relapse and treatment-related complications remain major clinical challenges. Accurate assessment of disease activity and relapse risk is critical for optimizing treatment duration and improving patient outcomes. In this context, noninvasive molecular imaging techniques have been explored as complementary tools for disease monitoring in AAV.

SSTR expression has been identified in inflammatory lesions associated with active AAV [39]. Immunohistochemical analyses have demonstrated the presence of SSTRs on various inflammatory cells within affected tissues, including macrophages, lymphocytes, and endothelial-like cells. Importantly, both granulomatous and non-granulomatous lesions exhibit detectable levels of SSTR expression, supporting its applicability across the spectrum of AAV subtypes. These findings provide the biological basis for the use of SSTR-targeted imaging to assess vascular inflammation and disease extent in AAV patients.

Neumann et al. conducted a study involving 32 patients with AAV, in which ^111^In-octreotide scintigraphy was performed at the time of initial diagnosis and during follow-up [39]. In selected cases, lung and mucosal biopsies were obtained for immunohistochemical evaluation of SSTR expression. The study found that SSTR scintigraphy had a specificity of 96 percent for detecting active pulmonary involvement and 100 percent for ear, nose, and throat (ENT) disease, with corresponding sensitivities of 86 percent and 68 percent. In patients who responded well to treatment, tracer uptake observed during the active phase diminished or disappeared on follow-up imaging. In contrast, persistent uptake was noted in patients with poor treatment response. Immunohistochemical analysis confirmed the presence of SSTR expression in biopsy samples from patients with both GPA and MPA. These results suggest that SSTR-based scintigraphy is useful not only for assessing disease activity and organ involvement, but also for monitoring therapeutic response in AAV. The presence of SSTRs across different histopathological forms of AAV further supports the potential utility of this imaging modality in routine clinical evaluation.

### 1.6. Large-Vessel Vasculitis

Large-vessel vasculitis (LVV) is a systemic inflammatory disorder that primarily affects large arteries, including the aorta and its major branches [40]. The two principal subtypes of LVV are giant cell arteritis (GCA) and Takayasu arteritis (TA). These conditions often present with nonspecific systemic symptoms and typically follow a relapsing-remitting course. If not promptly diagnosed and treated, they can lead to serious vascular complications such as arterial stenosis, occlusion, and aneurysm formation. While conventional imaging methods can detect structural vascular abnormalities, they may be limited in identifying early or subclinical inflammation. Molecular imaging techniques targeting SSTRs have emerged as promising tools for evaluating disease activity in LVV, particularly during the early phases or in patients with ambiguous clinical findings.

Ex vivo analyses have shown that SSTR2 is expressed on macrophages, pericytes, and perivascular adipocytes within affected vascular tissue [18]. These inflammatory cells frequently co-express proinflammatory markers, and SSTR2-specific ligand binding has been confirmed by autoradiographic techniques. These findings provide a biological rationale for the application of SSTR2-targeted radiotracers in molecular imaging of LVV, offering the potential to visualize active inflammation in the arterial wall with high specificity.

Corovic et al. conducted a prospective observational cohort study to evaluate the performance of SSTR2-targeted imaging using ^68^Ga-DOTATATE and ^18^F-FET-βAG-TOCA PET/MRI in patients with LVV [41]. The study enrolled 61 participants, including 27 patients with clinically diagnosed LVV, 25 individuals with recent myocardial infarction, and 9 oncologic controls. Arterial SSTR2 uptake, measured by maximum tissue-to-blood ratio (TBR), was significantly higher in patients with active or low-grade persistent inflammation compared to those with inactive disease. Specifically, the TBR in active LVV was 61.8 percent higher than in inactive disease and 34.6 percent higher than in myocardial infarction patients, with diagnostic performance yielding an area under the curve greater than 0.86. Notably, SSTR2 PET/MRI exhibited minimal physiological background uptake in the brain and heart, enabling clear visualization of adjacent vascular structures, including coronary, myocardial, and intracranial arteries.

In patients who received clinically effective treatment, follow-up imaging showed an average reduction of 22.3 percent in arterial SSTR2 TBR after approximately 9.3 months, suggesting the technique’s potential in monitoring therapeutic response. Ex vivo tissue analysis further confirmed that SSTR2 expression localized to inflammatory cells in vasculitic lesions, and specific receptor binding was demonstrated by autoradiography. These findings support the application of SSTR2-targeted PET/MRI as a promising modality for noninvasive assessment of disease activity and treatment response in patients with LVV.

Recently, a prospective study compared the imaging performance of ^68^Ga-HA-DOTA-TATE and ^18^FDG in eight patients with active giant cell arteritis (GCA) [42]. The results showed that ^18^FDG exhibited higher uptake in vascular lesions and was more sensitive for detecting inflammation. However, it demonstrated high background activity in the heart and was susceptible to interference from glucocorticoid therapy. In contrast, ^68^Ga-HA-DOTA-TATE showed significantly lower right atrial background signal, offering the potential for clearer visualization of inflammation in vascular regions adjacent to the heart and brain. Although this study did not demonstrate superiority of SSTR2 imaging over FDG in diagnostic accuracy, its potential utility in specific anatomical regions and patient subgroups warrants further investigation.

### 1.7. Thyroid Associated Ophthalmopathy

Thyroid-associated ophthalmopathy (TAO), also known as Graves’ ophthalmopathy, is the most common extrathyroidal manifestation of Graves’ disease [43]. It is characterized by orbital inflammation, extraocular muscle enlargement, and expansion of orbital adipose tissue, resulting in proptosis, diplopia, visual disturbances, and, in severe cases, vision loss and disfigurement. These complications significantly impair quality of life [44]. TAO occurs in approximately 25–50% of individuals with Graves’ disease, and 5–10% of them may develop clinically significant or sight-threatening manifestations. Although multiple therapeutic options are available, their efficacy is suboptimal, and only about two-thirds of patients experience meaningful clinical improvement. Since treatment response is closely linked to inflammatory activity, there is a pressing need for objective imaging techniques that can accurately assess disease activity and guide personalized treatment strategies.

SSTRs, particularly SSTR2, are expressed in activated lymphocytes and orbital fibroblasts in TAO [45,46]. In vitro studies have confirmed the expression of somatostatin and its receptor genes in primary fibroblast cultures derived from the retro-orbital tissues of patients with Graves’ ophthalmopathy [46]. These findings support the rationale for applying SSTR-targeted imaging in the evaluation of orbital inflammatory activity. Various radiotracers, including ^111^In-octreotide, ^99m^Tc-HYNIC-TOC, and ^99m^Tc-P829, have been developed to visualize SSTR expression in autoimmune and inflammatory conditions.

Krassas et al. performed ^111^In-Octreotide scintigraphy in 20 treated thyrotoxic patients with TAO, 5 treated thyrotoxic patients without TAO and 5 healthy individuals serving as controls prior to treatment with 300 micrograms of octreotide daily given for 12 weeks [47]. Seven patients showed an improvement if ocular manifestations, all of which proved positive on the initial scan whereas of those that did not respond only one was positive on the initial scan. Healthy controls also did not show uptake on the initial scan. This finding suggest that ^111^In-octreotide scintigraphy may allow for selection of those patients that may benefit from somatostatin therapy. Kahaly et al. compared orbital ^111^In-uptake in 45 patients with TAO and 10 control subjects [48]. In contrast to controls, patients presenting with TAO showed markedly increased orbital accumulation of ^111^In-octreotide.

In a study involving 46 patients with Graves’ ophthalmopathy and 4 healthy controls, ^99m^Tc-EDDA/HYNIC-TOC SPECT/CT was performed four hours after tracer administration to evaluate orbital inflammation [49]. The orbital-to-occipital (O/OC) uptake ratio was used as a quantitative measure. Among 35 patients who received oral corticosteroid therapy, 22 exhibited a significant decrease in O/OC ratio on follow-up imaging, corresponding with clinical improvement. In contrast, no significant changes were observed in the remaining 13 treated patients or in 9 of the 11 untreated patients. These results demonstrate the utility of ^99m^Tc-TOC SPECT/CT in evaluating disease activity and monitoring response to therapy, particularly in clinical settings where experience with subjective scoring systems such as the Clinical Activity Score (CAS) is limited.

A prospective study including 14 patients with moderate-to-severe TAO underwent ^99m^Tc-HYNIC-TOC scintigraphy prior to receiving retrobulbar radiotherapy [50]. Post-treatment evaluation at three months showed that 8 patients responded favorably, while 6 exhibited minimal or no clinical improvement. Pre-treatment O/OC ratios were significantly higher in responders (*p* = 0.001), and a positive correlation was noted between the O/OC ratio and CAS (*p* = 0.034). Receiver operating characteristic (ROC) analysis identified an O/OC threshold of 1.40 as optimal for distinguishing active from inactive disease, with 100% sensitivity and 83.3% specificity. All responders showed positive uptake, whereas 5 of the 6 non-responders had negative scans, suggesting that pre-therapeutic scintigraphy may aid in predicting therapeutic outcomes.

Burggasser et al. evaluated 44 patients with TAO using ^99m^Tc-P829 SPECT and planar scintigraphy, performed within three hours after injection [51]. The orbital-to-occipital (O/OCC) uptake ratio was significantly higher in patients with active disease compared to those with inactive TAO (1.69 ± 0.04 vs. 1.12 ± 0.05; *p* < 0.01). A strong correlation was observed between O/OCC ratio and CAS (r = 0.90), although no correlation was found with the NOSPECS classification or the superonasal index (SNI). Compared to ^111^In-labeled tracers, ^99m^Tc-P829 offers advantages in image quality, radiation safety, procedural efficiency, and cost, supporting its role as a practical imaging option for assessing disease activity in TAO.

Finally, more recently, Hu et al. studied 22 patients suffering from TAO and 6 healthy volunteers, all of whom underwent orbital ^68^Ga-DOTATATE PET/CT imaging. In line with the above referred studies, ^68^Ga-DOTATATE PET/CT proved to be a reliable method for assessing the inflammatory activity of extra-ocular muscles with an area under the curve exceeding 0.9 [52].

## 2. Discussion

SSTRs are a family of G-protein-coupled receptors (GPCRs) with five known subtypes (SSTR1–SSTR5), originally identified in neuroendocrine tissues [53]. SSTRs, particularly the SSTR2 subtype, have been increasingly recognized as potential molecular targets in the imaging of chronic inflammation and autoimmune diseases. Inflammatory microenvironments across a wide spectrum of conditions—such as sarcoidosis, vasculitis, autoimmune myocarditis, rheumatoid arthritis (RA), and thyroid-associated ophthalmopathy (TAO)—demonstrate upregulated expression of SSTRs on activated immune and stromal cells. These include M1 macrophages, CD4^+^T cells, dendritic cells, synovial fibroblasts, and orbital fibroblasts. This cellular distribution provides a strong biological foundation for SSTR-targeted molecular imaging as a non-invasive tool for assessing immune-mediated inflammation. As shown in Table 1, differences in SSTR subtype affinity among tracers (e.g., ^68^Ga-DOTATATE’s high selectivity for SSTR2, Kd ≈ 5 nM) may influence disease-specific imaging performance, particularly enhancing sensitivity in macrophage-dominant inflammation compared with broader-spectrum ligands such as ^68^Ga-DOTANOC.

The upregulation of SSTRs, particularly SSTR2 and SSTR5, is closely linked to pro-inflammatory signaling cascades. Cytokines such as tumor necrosis factor-α (TNF-α), interferon-γ (IFN-γ), and interleukin-1β (IL-1β) activate transcription factors including NF-κB and STAT1, which in turn drive the expression of SSTR genes in activated immune cells [54]. Among these, SSTR2 is most consistently expressed on classically activated (M1) macrophages, which are central to granulomatous and autoimmune inflammation. SSTR5 is also upregulated on certain T cell subsets and tissue-resident fibroblasts, contributing to chronic tissue damage and remodeling [55]. These molecular mechanisms explain the preferential accumulation of SSTR-targeted radiotracers at sites of active inflammation.

Radio-labeled somatostatin analogs—including ^68^Ga-DOTATATE, ^68^Ga-DOTANOC, ^99^ᵐTc-HYNIC-TOC, and ^111^In-pentetreotide—bind with high affinity to SSTR2 and related subtypes [56]. Upon receptor binding, these agents are internalized via receptor-mediated endocytosis, resulting in sustained intracellular retention and allowing for high-contrast imaging using PET or SPECT. This receptor-based targeting enables relatively specific visualization of inflamed lesions, while reducing nonspecific background uptake in normal tissues. By contrast, ^18^F-FDG accumulates in metabolically active tissues regardless of etiology, often leading to false positives in highly perfused or glucose-avid organs such as the brain, myocardium, and orbit [57]. In comparison, the widespread availability (>90% of centers supported) and high sensitivity (>85% in LVV/sarcoidosis) of ^18^F-FDG are endorsed by the 2024 EANM/SNMMI guidelines for FUO whole-body screening and glucocorticoid monitoring [58].

**Figure 1:**
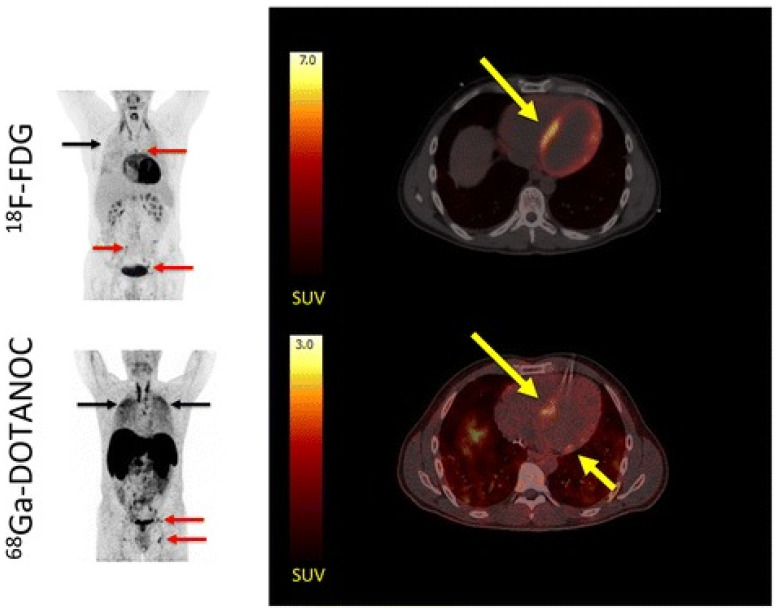
Comparative maximum intensity projection (MIP) and transaxial PET/CT images of ^18^F-FDG (**upper row**) and ^68^Ga-DOTANOC (**lower row**) in a patient with suspected cardiac sarcoidosis (adapted from Gormsen et al., 2016 [23], licensed under CC-BY 4.0). (**Left**) MIPs show avid hilar lymph node uptake (red arrows) and pulmonary parenchymal activity (black arrows) on both tracers, indicating systemic sarcoidosis involvement. (**Right**) Transaxial slices reveal focal-on-diffuse myocardial ^18^F-FDG uptake (SUV_max_ = 21; yellow arrow), which was rated inconclusive by most reviewers because of incomplete physiological suppression. In contrast, ^68^Ga-DOTANOC shows distinct focal septal uptake (SUV_max_ = 2.8; target-to-background = 3.0; yellow arrow), rated pathological by all reviewers. These images demonstrate the improved lesion conspicuity and reduced myocardial background achievable with SSTR-targeted imaging compared with FDG, facilitating diagnosis of cardiac involvement in sarcoidosis.

This distinction is particularly advantageous in diseases where physiological FDG uptake may mask pathological inflammation. In TAO, SSTR imaging permits accurate assessment of extraocular muscle inflammation, providing quantitative measures such as orbital-to-occipital uptake ratios that correlate with clinical activity and predict corticosteroid responsiveness [49]. Similarly, in atherosclerosis and sarcoidosis cardiac diseases, SSTR imaging—such as with ^68^Ga-DOTATATE or ^68^Ga-DOTANOC—has demonstrated potential advantages over ^18^F-FDG, offering improved specificity and image quality [12,23]. In atherosclerosis, ^68^Ga-DOTATATE and ^64^Cu-DOTATATE PET uptake was shown to correlate with plaque macrophage content, prior vascular events, and cardiovascular risk profiles. Compared to FDG, these tracers showed lower myocardial spillover and improved the visualization of inflamed vascular walls. In large-vessel vasculitis (LVV), including Takayasu arteritis and giant cell arteritis, SSTR PET/MRI provides reliable assessment of arterial wall activity, monitors treatment response, and offers a viable alternative in patients with inconclusive FDG scans [41]. This superior target-to-background ratio is further illustrated in Figure 1, which compares ^68^Ga-DOTATATE and ^18^F-FDG maximum-intensity projections from recent cardiac sarcoidosis cases [59]. Although SSTR-targeted imaging was initially performed using SPECT tracers such as ^111^In-pentetreotide and ^99^ᵐTc-EDDA/HYNIC-TOC, these agents have been largely replaced by PET counterparts, including ^68^Ga-DOTATATE, ^68^Ga-DOTATOC, and ^68^Ga-DOTANOC, owing to their superior spatial resolution, quantitative accuracy, and diagnostic sensitivity—particularly in neuroendocrine tumor imaging. Nevertheless, SPECT-based SSTR imaging remains clinically relevant in settings where PET systems or radionuclide generators are unavailable, offering a cost-effective and widely accessible alternative for the exploration of inflammatory diseases in resource-limited environments.

As summarized in Table 2, SSTR-targeted imaging generally demonstrates higher specificity and lower physiological background than ^18^F-FDG in selected inflammatory conditions. Nevertheless, FDG PET remains the reference standard for systemic inflammatory evaluation owing to its high sensitivity, established protocols, and global accessibility. FDG is particularly useful in fever of unknown origin (FUO) and large-vessel vasculitis (LVV), where comprehensive whole-body screening is required [58]. Therefore, SSTR-based imaging should be regarded as a complementary rather than a competitive technique, offering additional diagnostic value in cases where FDG is limited by high physiological uptake. As shown in Figure 1, SSTR-targeted PET using ^68^Ga-DOTANOC demonstrated clear focal uptake in the interventricular septum, whereas ^18^F-FDG imaging was confounded by physiological myocardial activity [23]. This example highlights the superior target-to-background contrast of SSTR imaging in cardiac sarcoidosis, consistent with findings summarized in Table 2.

**Table 2:** Comparative overview of SSTR- and FDG-targeted imaging in chronic inflammatory and immune-mediated diseases.

Disease	SSTR Imaging	FDG Imaging	Practical Notes	Authors
Atherosclerosis	uptake showed stronger association with vascular risk factors.	correlated weakly with hypertension only; limited overlap with SSTR uptake.	^68^Ga-DOTATATE may better reflect vascular macrophage activity and offers lower background in myocardium and brain.	LI et al. [12]
	showed strong uptake in macrophage-rich plaques and accurately identified culprit arteries.	uptake also correlated with inflammation but was limited by high myocardial background and poor coronary visualization.	^68^Ga-DOTATATE provided clearer coronary imaging and higher specificity for inflammatory plaques.	Tarkin et al. [13]
Cardiac sarcoidosis	with lower sensitivity, weak SSTR2 staining in granulomas	higher sensitivity, strong myocardial uptake	FDG preferred for cardiac inflammation; SSTR is useful for extra-cardiac lesions.	Bravo et al. [22]
	100% accuracy; moderate inter-observer agreement (κ = 0.46)	79% accuracy; poor agreement (κ = 0.27); many inconclusive scans despite fasting	SSTR imaging avoids myocardial uptake interference; promising alternative tracer for CS detection.	Gormsen et al. [23]
Large-Vessel Vasculitis	high specificity for vascular inflammation with low background in heart/brain, allowing clear visualization of arterial walls.	strong arterial uptake but suffers from high background and reduced accuracy under glucocorticoid therapy.	SSTR imaging offers superior visualization near myocardium or brain and potential for treatment monitoring, but FDG remains more sensitive in early disease.	Corovic et al. [41]
	lower arterial uptake and less conspicuous signal than FDG.	demonstrated higher vascular uptake and better lesion conspicuity.	FDG currently remains the preferred tracer; SSTR tracers may be useful when myocardial or cerebral artery involvement needs clearer assessment.	Clifford et al. [42]

Beyond the heart and vasculature, SSTR imaging has also proven valuable in pulmonary and extrapulmonary sarcoidosis, where it correlates with lymph node involvement and serum biomarkers of activity. In RA, SSTR SPECT imaging using ^99^ᵐTc-HYNIC-TOC enables visualization of active synovitis and may differentiate inflammatory from degenerative joint changes, offering utility in evaluating treatment response [35]. In ANCA-associated vasculitis (AAV), ^111^In-pentetreotide has shown potential in detecting ENT and pulmonary inflammation, although larger studies are needed to validate its diagnostic and prognostic accuracy [39].

Despite these advances, several limitations must be acknowledged. The expression of SSTRs, especially SSTR2, is heterogeneous across diseases and within different lesion types, which may reduce sensitivity in early-stage or low-grade inflammation [60]. Myocardial SSTR density is generally lower than in lymphoid or granulomatous tissues, limiting detection in subtle or patchy myocardial involvement [61]. Access to PET-based radiotracers such as ^68^Ga-DOTATATE may be restricted in some settings due to infrastructure requirements, while SPECT tracers, although more widely available, lack quantitative precision. Furthermore, the absence of standardized imaging protocols, uptake quantification thresholds, and interpretation criteria hinders cross-study comparability and routine clinical adoption. Finally, non-inflammatory SSTR expression in conditions such as neuroendocrine tumors or fibrotic tissue may contribute to false positives in certain scenarios. These challenges highlight the need for standardized methodology, multicenter validation, and integrated imaging strategies to maximize the clinical utility of SSTR-targeted imaging. Additionally, reference standards among included studies are heterogeneous. For instance, most myocarditis studies relied on MRI rather than endomyocardial biopsy (Table 3), with only one biopsy-based report confirming SSTR2 expression in limited samples (<20 patients). Similarly, atherosclerosis studies often lack histopathological validation, relying primarily on CT surrogates, which may underestimate SSTR efficacy [27].

**Table 3:** Summary of representative studies investigating SSTR-targeted molecular imaging in chronic inflammatory and immune-mediated diseases.

Disease	Author	Modality	Tracer	Setting	Reference Standard	Main Findings
**Atherosclerosis**	Rinne et al. [8]	PET	^68^Ga-DOTATATE and ^68^Ga-DOTANOC	Apolipoprotein E-deficient mice model	immunohistochemistry	Both tracers detected macrophage-rich plaques; ^68^Ga-DOTANOC showed higher vascular uptake.
	Meester et al. [9]	SPECT	^111^In-DOTA-JR11	ApoE^−^/^−^ mice and human carotid plaque samples	immunohistochemistry	High uptake in macrophage-rich plaques confirmed SSTR2 expression.
	Mojtahedi et al. [10]	PET	^68^Ga-DOTATATE	44 patients with neuroendocrine tumors	CT-based plaque classification (HU values) only	^68^Ga-DOTATATE showed increased uptake in atherosclerotic plaques compared with normal arteries.
	Rominger et al. [11]	PET	^68^Ga-DOTATATE	70 patients with neuroendocrine tumors	CT for calcified plaques only	^68^Ga-DOTATATE uptake correlated with calcified plaques, indicating potential for coronary plaque imaging.
	LI et al. [12]	PET	^68^Ga-DOTATATE and ^18^F-FDG	16 patients with neuroendocrine tumor or thyroid cancer	CT for calcified plaques only	^68^Ga-DOTATATE showed stronger correlation with vascular risk factors than ^18^F-FDG.
	Tarkin et al. [13]	PET	^68^Ga-DOTATATE and ^18^F-FDG	42 patients with atherosclerosis	CT and cardiovascular risk assessment only	^68^Ga-DOTATATE outperformed ^18^F-FDG in imaging macrophage-rich coronary plaques.
	Pedersen et al. [14]	PET	^64^Cu-DOTATATE	10 patients with carotid atherosclerosis	Gene expression	^64^Cu-DOTATATE uptake correlated with CD163 expression, indicating selective detection of M2 macrophages in symptomatic plaques.
	Malmberg et al. [15]	PET	^64^Cu-DOTATATE and ^68^Ga-DOTATOC	60 patients with neuroendocrine tumors	Cardiovascular risk factors only	^64^Cu-DOTATATE showed higher vascular uptake than ^68^Ga-DOTATOC and correlated with cardiovascular risk factors, suggesting potential for atherosclerosis assessment.
**Sarcoidosis**	Piotrowski et al. [19]	SPECT	^99m^Tc-HYNIC-TOC	32 patients with sarcoidosis	Biochemical and inflammatory markers	^99^ᵐTc-HYNIC-TOC uptake correlated with higher 8-isoprostane levels, suggesting value for assessing sarcoidosis activity.
	Kwekkeboom et al. [20]	SPECT	^111^In-pentetreotide	18 patients with biopsy-proven sarcoidosis	Chest X-ray, serum ACE, clinical course	^111^In-pentetreotide detected active granulomatous disease and correlated with higher ACE levels.
	Nobashi et al. [21]	PET	^68^Ga-DOTATOC Against ^67^Ga-scintigraphy	20 patients with histologically or clinically confirmed sarcoidosis	clinical diagnosis only	^68^Ga-DOTATOC PET/CT detected more lesions than ^67^Ga-scintigraphy and correlated with ACE levels, indicating superior sensitivity for active disease.
	Bravo et al. [22]	PET	^68^Ga-DOTATATE and ^18^F-FDG	13 patients with suspected CS	SSTR2 immunostaining	^68^Ga-DOTATATE was less sensitive than FDG for myocardial inflammation but comparable for extra-cardiac disease detection.
	Gormsen et al. [23]	PET	^68^Ga-DOTANOC and ^18^F-FDG	19 patients with suspected CS	Japanese Ministry of Health and Welfare (JMHW) CS criteria	^68^Ga-DOTANOC achieved 100% diagnostic accuracy and better inter-observer agreement than ^18^F-FDG PET/CT, which had many inconclusive scans.
	Lapa et al. [24]	PET	^68^Ga-DOTATATE	15 patients with systemic sarcoidosis and suspected cardiac involvement	Cardiac MRI only	^68^Ga-DOTATATE PET detected 7/15 cases; CMR positive in 10/15; overall concordance 96%, confirming feasibility and specificity for cardiac sarcoidosis imaging.
**Myocarditis**	Boursier et al. [28]	PET	^68^Ga-DOTATOC	14 patients with CMR-confirmed acute myocarditis	Cardiac MRI only	^68^Ga-DOTATOC uptake was elevated in all acute cases and persisted in some at 4 months, suggesting sensitivity to residual inflammation.
	Larive et al. [29]	PET	^68^Ga-DOTATOC	31 patients with acute myocarditis	Cardiac MRI only	^68^Ga-DOTATOC uptake was elevated in all AM cases; quantitative thresholds differentiated AM from nonspecific uptake with 87–94% accuracy.
	Aparici et al. [30]	SPECT	^111^In-pentetreotide	10 heart transplant recipients	Endomyocardial biopsy	^111^In-pentetreotide uptake (HLR > 1.6) correlated with active or impending rejection, preceding biopsy positivity by 1 week.
**Rheumatoid arthritis**	Anzola et al. [35]	SPECT	^99m^Tc-EDDA/HYNIC-TOC	18 patients with RA and Sjögren’s syndrome	Clinical evaluation	^99^ᵐTc-EDDA/HYNIC-TOC uptake decreased in joints but not salivary glands after anti-TNFα therapy, reflecting treatment response and disease extent.
	Vanhagen et al. [36]	SPECT	^111^In-octreotide	14 patients with RA	Clinical findings, histologic synovial analysis	76% of painful/swollen joints showed ^111^In-octreotide uptake; SS-R expression confirmed in RA synovium but minimal in OA or controls.
**ANCA**	Neumann et al. [39]	SPECT	^111^In-octreotide	32 patients with AASV	Clinical activity assessment and histology	^111^In-octreotide accurately identified active pulmonary/ENT lesions and reflected treatment response in AASV.
**Large-vessel Vasculitis**	Corovic et al. [41]	PET	^68^Ga-DOTATATE	27 patients with clinically diagnosed LVV	Clinical activity and histopathology	^68^Ga-DOTATATE detected active LVV with high accuracy (AUC ≥ 0.86) and low background; signal decreased after treatment and localized to proinflammatory macrophages.
	Clifford et al. [42]	PET	^68^Ga-HA-DOTA-TATE and ^18^FDG	8 patients with active giant cell arteritis	Visual uptake grading and quantitative	^68^Ga-HA-DOTATATE showed lower arterial uptake than ^18^F-FDG, with no clear advantage for vascular inflammation detection.
**TAO**	Krassas et al. [47]	SPECT	^111^In-Octreotide	20 treated thyrotoxic patients with TAO	Clinical response to octreotide	Positive scans predicted favorable therapeutic response to octreotide.
	Kahaly et al. [48]	SPECT	^111^In-octreotide	45 patients with TAO	Clinical diagnosis	Significantly increased orbital uptake in TAO compared with controls.
	Zhao et al. [49]	SPECT	^99m^Tc-EDDA/HYNIC-TOC	46 patients with Graves’ ophthalmopathy	Clinical activity score	O/OCC ratio strongly correlated with CAS (r = 0.90).
	Sun et al. [50]	SPECT	^99m^Tc-HYNIC-TOC	14 patients with moderate-to-severe TAO	Response to radiotherapy	Pre-treatment O/OC > 1.4 predicted good radiotherapy response.
	Burggasser et. [51]	SPECT	^99m^Tc-P829	44 patients with TAO	Clinical follow-up	Decrease in O/OC ratio correlated with clinical improvement.
	Hu et al. [52]	PET	^68^Ga-DOTATATE	22 patients suffering from TAO	MRI and clinical grading	Accurately assessed EOM inflammation (AUC > 0.9).

Note: Only representative studies explicitly discussed in the text are included.

Looking forward, several strategies may enhance the role of SSTR imaging in immune-mediated diseases. Combining SSTR-targeted imaging with radiomic features and artificial intelligence algorithms could improve detection sensitivity, pattern classification, and response prediction. Dual-tracer protocols or the integration of SSTR imaging with other inflammation-targeted agents—such as CXCR4, TSPO, or FAPI—may provide complementary insights into immune cell composition and fibrotic remodeling [62]. Furthermore, the development of theranostic SSTR ligands opens avenues for targeted therapy delivery in refractory inflammatory conditions.

In parallel, novel PET tracers targeting non-metabolic inflammatory pathways have expanded the landscape of molecular imaging beyond ^18^F-FDG. Among them, fibroblast activation protein inhibitors (FAPI, e.g., ^68^Ga-FAPI-04) visualize fibroblast activation and fibrosis in disorders such as systemic sclerosis, IgG4-related disease, and Crohn’s disease, enabling better distinction between fibrotic and inflammatory lesions [63,64]. CXCR4 ligands (e.g., ^68^Ga-pentixafor) map chemokine-driven immune cell recruitment in rheumatoid arthritis, post-infarction remodeling, and atherosclerosis, offering potential prognostic value for cardiac recovery and disease progression [65,66]. RGD peptides (e.g., ^18^F-fluciclatide) bind αvβ3 integrin expressed on angiogenic endothelium, highlighting neovascularization within plaques, synovial tissue, and ischemic lesions [67]. Collectively, these emerging tracers enable precision phenotyping of inflammatory diseases, facilitating personalized treatment strategies and objective monitoring of therapeutic efficacy. These tracers complement SSTR (e.g., FAPI in fibrotic sarcoidosis), enhancing inflammation subtyping.

This review has some limitations. First, the reference standards across the included studies are heterogeneous, ranging from histopathology and immunohistochemistry to clinical diagnosis and imaging surrogates. This variability hampers direct comparison of diagnostic performance and may affect the strength of evidence supporting SSTR imaging in specific diseases. Second, most available studies are single-center investigations with small sample sizes and diverse imaging protocols, precluding robust statistical conclusions or generalization to broader clinical populations. Future research should therefore focus on prospective, multicenter trials with standardized acquisition and interpretation criteria to better define the diagnostic and prognostic value of SSTR-targeted imaging across different inflammatory contexts.

In conclusion, SSTR-targeted molecular imaging appears to be a biologically grounded, technically feasible, and potentially useful approach for characterizing inflammation across a range of autoimmune and chronic inflammatory disorders. While FDG PET remains the gold standard, SSTR imaging provides complementary advantages in specificity and lesion localization, particularly in high-background organs such as the brain and myocardium. Continued standardization, multicenter validation, and integration with advanced analytic methods will be crucial for establishing its role in precision diagnostics and individualized care.

## Figures and Tables

**Table 1:** Molecular characteristics and receptor-binding selectivity of SSTR-targeted PET and SPECT tracers in chronic inflammatory and immune-mediated diseases.

Radionuclide	Half-Life	Tracer	SSTR Subtype
^68^Ga	1.13 h	^68^Ga-DOTATATE	SSTR2
		^68^Ga -DOTANOC	SSTR2, SSTR3, SSTR5
		^68^Ga -DOTATOC	SSTR2>> SSTR5
^64^Cu	12.7 h	^64^Cu-DOTATATE	SSTR2
^111^In	67.2 h	^111^In-pentetreotide	SSTR2
		^111^In-octreotide	SSTR2
		^111^In-DOTA-JR11	SSTR2> SSTR5
^99m^Tc	6 h	^99m^Tc-HYNIC-TOC	SSTR2, SSTR5
		^99m^Tc-P829	SSTR2, SSTR3, SSTR5
